# Victimization of Peruvian adolescents and health risk behaviors: young lives cohort

**DOI:** 10.1186/1471-2458-14-85

**Published:** 2014-01-28

**Authors:** Benjamin T Crookston, Ray M Merrill, Stephanie Hedges, Cameron Lister, Joshua H West, P Cougar Hall

**Affiliations:** 1 Department of Health Science, College of Life Sciences, Brigham Young University, 229-G Richards Building, Provo, Utah 84602, USA

**Keywords:** Alcohol, Bullying, Caregiver, Children, Sexual relations, Smoking, Victimization

## Abstract

**Background:**

While extensive research has been conducted on bullying and victimization in western countries, research is lacking in low- and middle-income settings. This study focused on bullying victimization in Peru. It explored the relationship between the caregiver’s perception of child victimization and the child’s view of selected negative experiences occurring with other children their age. Also, the study examined the association between victimization and adolescent health risk behaviors.

**Methods:**

This study used data from 675 children participating in the Peru cohort of the Young Lives study. Children and caregivers were interviewed in 2002 when children were 8 years of age and again in 2009 when children were 15 years of age. Measures of victimization included perceptions from children and caregivers while measures of health risk behaviors included cigarette smoking, alcohol drinking, and sexual relations among adolescents.

**Results:**

Caregivers identified 85 (12.6%) children bullied at ages 8 and 15, 235 (34.8%) bullied at age 8 only, 61 (9.0%) bullied at age 15 only, and 294 (43.6%) not bullied at either age. Children who were bullied at both ages compared with all other children were 1.58 (95% CI 1.00-2.50) times more likely to smoke cigarettes, 1.57 (1.04-2.38) times more likely to drink alcohol, and 2.17 (1.41-3.33) times more likely to have ever had a sexual relationship, after adjusting for gender. The caregiver’s assessment of child victimization was significantly associated with child reported bullying from other children their age. Child reported victimization was significantly associated with increased risky behaviors in some cases.

**Conclusion:**

Long-term victimization from bullying is more strongly associated than less frequent victimization with increased risk of cigarette smoking, alcohol drinking, and sexual relations at age 15. Hence, programs focused on helping children learn how to mitigate and prevent bullying consistently over time may also help reduce risky adolescent health behaviors such as smoking, alcohol consumption, and sexual activity.

## Background

Bullying during childhood and adolescence is a common experience that has potentially far reaching negative implications [1-3]. Bullying is a broad construct that is manifested by deliberate antagonistic behavior by the bully towards the victim, persistence of this behavior over a period of time, and an imbalance of power between the bully and the victim [4-8]. Bullying is often characterized as either direct—personal confrontation between the bully and the victim—or indirect—passive interaction such as social exclusion, spreading rumors, and cyber-bullying [8-13].

Bullying and victimization among children are associated with high-risk health behaviors such as substance abuse, sexual activity, and suicidal ideation and attempts [14-16]. In a study focusing on middle and high school youth, substance abuse (alcohol, cigarettes, and marijuana) was greater among both those perpetuating the bullying and the victims, with greater use occurring among victims [14]. Earlier studies have also found an association between bullying and substance abuse [17-19]. Recent associations between bullying and increased sexual activity have similarly been identified [20,21].

Bullying is a global problem, with between 10 and 30 percent of youth involved in some aspect of bullying (bully and/or victim) [5]. High levels of bullying exist throughout the U.S. [19,22,23], Europe [24,25], Australia [26], and Latin America [27-29]. Data from the 2005 Health Behavior in School-aged Children survey, a representative sample of grades 6-10 in the United States, showed that bullying in the past two months was commonly done through direct and indirect ways: physical (21%), verbal (54%), social (51%), and electronically (14%) [23].

Bullying and victimization is less understood in low- to middle-income countries due to less available data. In several Latin American countries, early studies show the prevalence of bullying to be as high as 50%. In Peru and Colombia, for example, bullying among teens may involve as much as half of the population [28-32]. Studies show that bullying among Peruvian adolescents is both physical and psychological, involves more males than females, and is most frequent between the ages of 10-16 [33,34]. In 50 cities throughout Peru, researchers found that of the students who reported being bullied, 54.4% were bullied verbally, 35.9% were involved in physical aggression, 26.7% were excluded from groups, and 12.8% were bullied by mixed forms of violence [32]. A 2008 study reported that 47% of Peruvian teenagers were bullied [28]. However, this study noted that a third of the students did not tell a parent or a teacher of the bullying incident, indicating that the level of bullying may be much higher and that caregivers may largely be unaware of the extent of this problem.

The current study focused on bullying victimization among a Peruvian cohort. It explored the relationship between the caregiver’s perception of child bullying, how this perception related to the child’s view of selected negative experiences with other children their age, and how these experiences related to cigarette smoking, alcohol drinking, and sexual relations. Specifically, the study examined if the caregiver’s assessment of bullying at ages 8 and 15 associated with increased risk of current cigarette smoking and alcohol drinking and ever having had sexual relations for children aged 15 years. The study also evaluated whether child-specific items involving negative associations with other children of a similar age were related to the caregiver’s assessment of their child being bullied. Lastly, the study assessed whether child-specific items involving negative associations with other children of a similar age were related to cigarette smoking, alcohol drinking, and having ever had sexual relations among children aged 15 years.

## Methods

### Young lives

Young Lives is an international prospective study of childhood poverty, education, and health among approximately 12,000 children in four countries: Ethiopia, India, Peru, and Vietnam. A detailed description of the study design, methods for sampling, recruitment, and interviewing are described in detail elsewhere [35]. In brief, the Young Lives study followed two cohorts of children in each country, a younger cohort of 2,000 children who were recruited at approximately 1 year of age and an older cohort of approximately 1,000 children enrolled at 8 years of age. This study focused on data from the older Peruvian cohort.

Peruvian children were randomly selected from 74 communities within 20 random districts comprising the poorest 95% of districts in Peru. Children and caregivers were interviewed at enrollment (Round 1), in 2006 (Round 2), and again in 2009 (Round 3) when children were ages 8, 12, and 15, respectively. Fieldworkers were trained extensively prior to data collection to standardize data collection protocols. This study used data from 675 children and caregivers for whom data was available when the child was 8 and 15 years of age.

### Measures

The caregiver’s assessment of bullying is based on the question: “Is NAME, picked on or bullied by other children?” This question was asked at ages 8 and 15. For part of the analysis, the association between the combinations of bullying at the two ages (yes-yes, yes-no, no-yes, and no-no) with cigarette smoking, alcohol drinking, and sexual relations were evaluated. At age 15, eight child-specific items involving negative associations with other children their age were asked to the children. They were told to indicate their level of experience with each of these items: never, once, 2-3 times, or 4+ times. In some cases, responses were dichotomized as never/once and 2+ times to improve analytical power. Questions about current cigarette smoking, alcohol drinking, and whether they had ever had a sexual relationship were asked to the children at age 15.

### Ethics

Ethical approval for the Young Lives study was granted by London South Bank University, the London School of Hygiene and Tropical Medicine, the University of Reading and from the IIN. Approval for this study was granted by the Institutional Review Board of Brigham Young University. Only households that provided informed consent were included.

### Statistical techniques

Incidence rates were used to summarize the data and describe distributions. The assessment of bullying at ages 8 and 15 by caregivers were evaluated using McNemar’s chi-square test. Independence between categorical variables was evaluated for statistical significance using the chi-square test. The Mantel-Haenszel chi-square was used to evaluate trends. Relative risks were calculated to compare incidence between groups. These ratios were adjusted for gender and evaluated for statistical significance using 95% confidence intervals. Three multivariate regression analyses were conducted involving child-perceived negative behavior variables regressed on cigarette smoking, alcohol drinking, and then sexual relations, adjusted for gender. Each of these models was significant based on Wilks’ Lambda, indicating that it was appropriate to do bivariate analyses on these variables. Finally, multiple Poisson regression was used to simultaneously assess the significance of a combination of child-perceived negative behavioral items on caregiver assessment of bullying, current cigarette smoking, current alcohol drinking, and ever having had a sexual relationship. Two-sided tests of hypotheses were evaluated using the 0.05 level of significance. Analyses were performed using the Statistical Analysis System (SAS) software, version 9.3 (SAS Institute Inc., Cary, NC, USA, 2010).

## Results

Bullying status, as reported by the caregiver, significantly changed from age 8 (320, 47.4%) to age 15 (146, 21.6%) based on the McNemar test (p < 0.0001). The combination of bullying status at ages 8 and 15 is shown in Table 1. At age 15, questions were first asked about the children’s smoking, alcohol, and sexual behaviors, wherein 18.1% (26.2% of males and 10.9% of females, p < 0.0001) smoked cigarettes, 36.6% (37.5% for males and 36.3% for females, p = 0.7299) drank alcohol, and 24.5% (31.4% for males and 17.4% for females, p < 0.0001) had engaged in sexual relations. Children who caregivers reported were bullied at both ages 8 and 15 had a significantly greater level of smoking, drinking, and past sexual experience at age 15. Children bullied at age 8 but not age 15, or not at age 8 but at age 15, were less likely to smoke cigarettes, drink alcohol, or have had a sexual relationship. Children who were bullied at both ages 8 and 15 compared with the other groups of children, adjusting for gender, were 1.58 times more likely to smoke cigarettes, 1.57 times more likely to drink alcohol, and 2.17 times more likely to have had a sexual relationship.

**Table 1:** Bivariate analyses of bullying status at ages 8 and 15 according to current smoking, alcohol drinking, and having had sex at age 15

	**Yes–Yes n = 85 (12.6%)**	**Yes–No n = 235 (34.8%)**	**No–Yes n = 61 (9.0%)**	**No–No n = 294 (43.6%)**	**Chi-square/P value**	Relative risk^*^	**95% CI**
Cigarette smoking							
Yes	29.0	17.8	17.2	17.4	5.66	1.58^†^	1.00–2.50
No	71.0	82.2	82.8	82.6	0.13	1.00	
Alcohol drinking							
Yes	48.0	33.8	29.3	38.0	6.62	1.57^†^	1.04–2.38
No	52.0	66.2	70.7	62.0	0.09	1.00	
Sexual relations							
Yes	42.9^†^	23.7^†^	20.7^†^	21.6^†^	15.68	2.17^†^	1.41–3.33
No	57.1	76.3	79.3	78.4	0.00	1.00	

^*^Yes–Yes vs. otherwise, adjusted for gender.

^†^p < 0 · 05.

Notes: Incidence of caregiver report of child bullied at ages 8 and 15: Yes-Yes (bullied at age 8 and 15), Yes-No (bullied at age 8, but not 15), No-Yes (not bullied at age 8, but bullied at age 15), and No-No (not bullied at age 8 or 15).

More than half (59.4%) of children reported having others stealing something from them at age 15 (Table 2). The least common forms of child-reported negative experiences at age 15 were physical harm or damaging one’s property. Using multiple Poisson regression to test for agreement between caregiver report of bullying and child-reported bullying, model estimates indicated that three of the eight child-reported items were significantly associated with caregiver report of child being bullied at age 15 and at both ages 8 and 15: “Tried to get you into trouble with your friends,” “Made fun of you for some reason,” and “Punched, kicked, or beat you up” (data not shown). Caregiver report of child being bullied at both ages 8 and 15 compared with those not bullied at either age were significantly more likely to be punched, kicked or beat up at age 15 (Relative Risk = 2.52, 95% CI = 1.40-4.53). Those bullied at age 8 but not at age 15 compared with not bullied at either age were not significantly more likely to experience any of the items in Table 2. In a third model, those not bullied at age 8 but bullied at age 15 compared with not bullied at either age were significantly more likely to have someone try to get them in trouble with their friends (Relative Risk = 1.95, 95% CI = 1.27-2.99). All three models adjusted for gender.

**Table 2:** Frequency of child-reported selected bullying-related items at age 15

		**Frequency**			
		**(Never)**	**(Once)**	**(2-3 Times)**	**(4+ Times)**
**Have other young people …**	**No.**	**%**	**%**	**%**	**%**
Taken something without permission or stolen from you	610	40.6	30.5	17.6	11.3
Tried to get you into trouble with your friends	608	48.5	34.1	11.8	5.6
Made fun of you for some reason	617	52.4	33.1	9.2	5.3
Refused to talk to you or made other people not talk to you	616	57.1	28.1	8.4	6.4
Made you uncomfortable by staring at you for a long time	617	58.2	26.6	11.8	3.4
Tried to break or damage something of yours	612	61.2	27.4	8.5	2.9
Punched, kicked or beat you up	614	83.2	12.5	2.8	1.5
Hurt you physically in any other way	614	73.3	20.0	4.4	2.3

Multivariate regression analysis was conducted with each of the eight variables of child-reported negative experience, simultaneously regressed on the cigarette smoking variable, adjusted for gender, which gave a Wilks’ Lambda p = 0.0478. Similar models were run for alcohol (< 0.0001), and sexual relations (0.0180). Relative risks adjusted for gender are presented for each of the eight items in Table 3.

**Table 3:** Bivariate analyses of selected behaviors according to child-specific items involving negative experiences with peers

	**Cigarette smoking**		**Alcohol drinking**		**Sexual relations**	
**Have other young people …**	Relative risk^*^	**95% CI**	Relative risk^*^	**95% CI**	Relative risk^*^	**95% CI**
Taken something without permission or stolen from you	0.81	0.54–1.20	1.22	0.98–1.51	1.19	0.88–1.62
Tried to get you into trouble with your friends	1.59^†^	1.09–2.30	1.87^†^	1.52–2.30	1.76^†^	1.30–2.38
Made fun of you for some reason	1.15	0.74–1.77	1.45^†^	1.14–1.85	1.55^†^	1.12–2.15
Refused to talk to you or made other people not talk to you	1.05	0.66–1.69	1.40^†^	1.09–1.79	1.64^†^	1.18–2.30
Made you uncomfortable by staring at you for a long time	1.17	0.75–1.82	1.35^†^	1.06–1.73	1.51^†^	1.08–2.12
Tried to break or damage something of yours	0.93	0.55–1.59	1.19	0.88–1.61	1.34	0.90–1.98
Punched, kicked or beat you up	2.13^†^	1.31–3.48	1.39	0.93–2.08	2.00^†^	1.30–3.09
Hurt you physically in any other way	1.71^†^	1.07–2.74	1.40^†^	1.01–1.95	2.01^†^	1.38–2.91

^*^Each of the eight items was dichotomized as 2+ times vs. none/one time, adjusted for gender.

^†^p < 0 · 05.

The incidence of cigarette smoking, alcohol drinking, and sexual relations was associated with a number of child-specific items involving negative experiences with other children their age. Two key items, “Tried to get you in trouble with your friends” and “Hurt you physically in any other way” were significantly positively associated with cigarette smoking, alcohol drinking, and sexual relations. The incidence of alcohol drinking and sexual relations were additionally associated with items measuring being made fun of, refusing to talk to, and made uncomfortable by staring at you for a long time. “Taken something without permission or stolen from you” or “Tried to break or damage something of yours” were not significantly associated with cigarette smoking, alcohol drinking, or sexual relations.

Using multiple Poisson regression, two items, “Tried to get you in trouble with your friends” and “Punched, kicked or beat you up”, as well as gender, were consistently associated with both cigarette smoking and sexual relations (Table 4). The first item was also associated with drinking alcohol. Interaction terms involving the two items were not significant in any of the models.

**Table 4:** Multiple Poisson regression assessing selected behaviors according to bullying-related items at age 15

	**Cigarette smoking at age 15**		**Alcohol drinking at age 15**		**Ever had sexual relations by age 15**	
**Have other young people …**	**Relative risk**	**95% CI**	**Relative risk**	**95% CI**	**Relative risk**	**95% CI**
Tried to get you into trouble with your friends	1.54^*^	1.05–2.25	1.86^*^	1.51–2.31	1.69^*^	1.25–2.30
Punched, kicked or beat you up	1.98^*^	1.21–3.25	1.25	0.86–1.83	1.79^*^	1.17–2.75
Gender (M vs. F)	2.58^*^	1.73–3.84	1.05	0.85–1.29	1.80^*^	1.32–2.45

*p < 0.05.

Notes: The two bullying-related items were categorized as 2+ times vs. none/one time. The relative risks were simultaneously estimated, with all three variables in each model.

## Discussion

This study addressed three specific research questions involving bullying victimization and substance use among children in Peru. The first question explored whether the caregiver perceived bullying at both ages 8 and 15 was associated with increased risk of cigarette smoking, alcohol drinking, and having ever had sexual relations for children aged 15 years. Although previous research has shown that bullying victimization is associated with increased risk for addictive behaviors, such as smoking cigarettes and drinking alcohol [36,37], this study found that only children identified by caregivers as being bullied at both ages 8 and 15 were significantly more likely to smoke cigarettes, drink alcohol, and have had sexual relations at age 15. Those who were bullied at just age 8 or just age 15 had levels of smoking, drinking and sexual relations similar to those who had never been bullied. This indicates that long-term bullying is more predictive of substance use and sexual relations. While preventing bullying entirely remains a primary objective, this finding highlights the benefit and importance of limiting the duration of bullying to a short-term and infrequent occurrence. Perhaps most importantly, this finding provides substantial hope that mediating interventions targeted at those who have experienced bullying in the short-term can help prevent adolescent health risk behaviors.

The second research question explored whether child-specific items involving negative associations with other children of a similar age were related to the caregiver’s assessment of their child being bullied. Indirect forms of bullying victimization were more common than direct forms [8-13]. Increasing frequency of child-reported negative experiences was associated with caregiver indication of child bullying for the items: “Tried to get you into trouble with your friends,” “Made fun of you for some reason,” and “Punched, kicked, or beat you up.” “Hurt you physically in any other way” was marginally insignificant. This is generally consistent with previous research demonstrating that while a majority of children do tell their parents when they are bullied, a substantial amount (roughly 1 in 3) do not and that children who were bullied more frequently were more likely to tell their parents [38]. Hence, while caregiver perceptions of peer-victimization is a good predictor of actual child victimization, not all children tell their parents, particularly those who experience less peer victimization. These findings demonstrate a need for empowering children with improved decision-making and communication skills while encouraging them to speak with a trusted adult or caregiver whenever bullying occurs. Additionally, these findings highlight the challenge for researchers in identifying additional measures for both identifying and quantifying early childhood bullying. Further, this research indicates a gap between various types of child-reported victimization and victimization recognized by caregivers. Establishing better measures of bullying behaviors together with helping adults and children accurately define and recognize bullying behaviors provides promise in narrowing this gap [39]. Future efforts might also be directed at improving communications between children and caregivers regarding the topic of bullying victimization.

This study also assessed whether the child-specific items involving negative associations with other children of a similar age related to current cigarette smoking, alcohol drinking, and having ever had sexual relations among children aged 15 years. One study found that all forms of bullying increases the risk of smoking cigarettes and drinking alcohol [40]. This differs from the findings of the current study which demonstrated that long-term bullying associated more with smoking, drinking, and sexual relations, and that only “Tried to get you into trouble with your friends” and “Punched, kicked or beat up” had independent direct effects on smoking and sexual relations with the first item also associated with alcohol drinking. Although other research among children in third grade has found that experiencing both relational and physical aggression had the greatest risk for maladjustment a year later [20], the models utilized in the current study did not find a similar interaction effect. Although slight variations in associations among bullying measures and behaviors exist between previous research and the current study, the current findings strongly support the association between bully victimization and adolescent health risk behaviors.

Children try to fit in and be well-liked by their peers. One study observed that victims of bullying are more likely to become bullies themselves [27]. Perhaps this is an attempt to fit in and be accepted. Along the same lines, victims of bullying may be more likely to turn to smoking, alcohol drinking, or sexual activity as a way to be accepted by peers. Turning to these health risk behaviors during adolescence may be a coping strategy employed to deal with their victimization.

This study has several limitations. Young Lives is a study of poverty and was not designed to explore the correlates of bullying in-depth. However, Young Lives does contain a richness of data collected longitudinally that is not typically available in low-resource settings. Further, while this study has multiple time points over adolescence, much is not known about what happens to the child between measurement periods. As a result, this study provides important information on the possible pathways that connect bullying and risky behaviors, but falls short of constructing a complete picture.

## Conclusion

In summary, persistent bullying across time is associated with increased adolescent health risk behaviors compared to those who experienced bullying only at one time or not at all. Hence, programs focused on teaching children how to mitigate and prevent bullying consistently over time may also help reduce adolescent health risk behaviors such as smoking, alcohol consumption, and sexual activity. Further, this research demonstrates that while there is need for improvement, caregivers are generally attuned to specific child victimization experiences. However, more research is needed to understand how children make decisions on when to disclose such experiences to caregivers and how to increase and improve such communication.

## Abbreviations

CI: Confidence interval; MH: Mantel-Haenszel.

## Competing interests

The authors declare that they have no competing interests.

## Authors’ contributions

BC acquired the data and BC and RM conceived the design of the study. RM completed the analysis and wrote the initial draft. All authors contributed to the writing and approved the final manuscript.

## Authors’ information

BC, JW, and PH are assistant professors in the Department of Health Science at Brigham Young (BYU) University. RM is a professor in the Department. SH is an undergraduate student in Public Health at BYU and CL is a Master of Public Health Student at BYU.

## Pre-publication history

The pre-publication history for this paper can be accessed here:

http://www.biomedcentral.com/1471-2458/14/85/prepub
